# Challenges Facing Airway Epithelial Cell-Based Therapy for Cystic Fibrosis

**DOI:** 10.3389/fphar.2019.00074

**Published:** 2019-02-08

**Authors:** Andrew Berical, Rhianna E. Lee, Scott H. Randell, Finn Hawkins

**Affiliations:** ^1^Center for Regenerative Medicine, Boston Medical Center and Boston University, Boston, MA, United States; ^2^The Pulmonary Center, Boston University School of Medicine, Boston, MA, United States; ^3^Cystic Fibrosis Research Center, Marsico Lung Institute, University of North Carolina at Chapel Hill, Chapel Hill, NC, United States; ^4^Department of Cell Biology and Physiology, University of North Carolina at Chapel Hill, Chapel Hill, NC, United States

**Keywords:** cystic fibrosis, cell-based therapy, induced pluripotent stem cells, human bronchial epithelial cells, engraftment

## Abstract

Mutations in the cystic fibrosis transmembrane conductance regulator (*CFTR*) gene cause the life-limiting hereditary disease, cystic fibrosis (CF). Decreased or absent functional CFTR protein in airway epithelial cells leads to abnormally viscous mucus and impaired mucociliary transport, resulting in bacterial infections and inflammation causing progressive lung damage. There are more than 2000 known variants in the *CFTR* gene. A subset of CF individuals with specific *CFTR* mutations qualify for pharmacotherapies of variable efficacy. These drugs, termed CFTR modulators, address key defects in protein folding, trafficking, abundance, and function at the apical cell membrane resulting from specific *CFTR* mutations. However, some *CFTR* mutations result in little or no CFTR mRNA or protein expression for which a pharmaceutical strategy is more challenging and remote. One approach to rescue CFTR function in the airway epithelium is to replace cells that carry a mutant *CFTR* sequence with cells that express a normal copy of the gene. Cell-based therapy theoretically has the potential to serve as a one-time cure for CF lung disease regardless of the causative *CFTR* mutation. In this review, we explore major challenges and recent progress toward this ambitious goal. The ideal therapeutic cell would: (1) be autologous to avoid the complications of rejection and immune-suppression; (2) be safely modified to express functional CFTR; (3) be expandable *ex vivo* to generate sufficient cell quantities to restore CFTR function; and (4) have the capacity to engraft, proliferate and persist long-term in recipient airways without complications. Herein, we explore human bronchial epithelial cells (HBECs) and induced pluripotent stem cells (iPSCs) as candidate cell therapies for CF and explore the challenges facing their delivery to the human airway.

## Introduction

Cystic fibrosis (CF) is an autosomal recessive, multisystem, genetic disease caused by mutations in the cystic fibrosis transmembrane conductance regulator (*CFTR)* gene resulting in deficient and/or defective CFTR protein (Cutting, 2014; Ratjen et al., 2015). CFTR is an anion channel present across a number of epithelia including the lungs, intestine, sinuses, pancreas, biliary tree, and vas deferens. The consequences of CFTR dysfunction are pronounced in the lungs where ineffective chloride and bicarbonate ion transport results in an abnormally viscous and acidic apical surface layer (ASL). This abnormal environment is colonized by bacteria in early life and a cycle of infection and inflammation results in bronchiectasis and end-stage lung disease (Ratjen et al., 2015). Disease severity is determined to a large extent by the causative *CFTR* mutation(s). Over 2,000 variants in *CFTR* have been described, of which approximately 300 have been determined to be pathogenic (cftr2.org). These variants or combinations of variants have differing effects on the amount and function of CFTR protein. Some variants are associated with milder disease or particular organ involvement while others may be associated with very severe disease. For classification purposes, these mutations are grouped into six classes (I-VI) based on their effect on CFTR including: no protein synthesis (class I), protein misfolding (class II), dysfunctional channel gating (class III), reduced conductance (class IV), insufficient CFTR protein due to abnormal RNA splicing (class V), or increased protein turnover (class VI).

Mucus clearance techniques, antibiotics, and lung transplantation significantly improve the life expectancy of CF individuals. The recent discovery of CFTR modulators has ushered in a new era of precision medicine for CF patients with mutations that result in some residual druggable CFTR protein. For example, the major defect in patients with the class III mutation G551D is diminished channel activity at the apical surface. Ivacaftor is an FDA approved CFTR potentiator that increases CFTR activity and results in clinical improvement in patients with at least one copy of the G551D mutation (Ramsey et al., 2011). F508del is the most common CFTR mutation affecting approximately 90% of CF patients (Cutting, 2014). This mutation results in defective folding and trafficking of the CFTR protein. Corrector molecules such as lumacaftor and tezacaftor in conjunction with ivacaftor result in increased CFTR activity and some clinical improvement though not as robust as the response of gating and residual function mutations to ivacaftor therapy (Rowe et al., 2017; Taylor-Cousar et al., 2018). Recent progress with “triple combination” regimens including two correctors plus the potentiator ivacaftor indicates increased efficacy for those harboring the class II F508del mutation (Davies et al., 2018; Keating et al., 2018). Class I mutations are non-sense, frame-shift or splice variants that result in premature termination of the CFTR transcript and no CFTR protein. These patients currently have no targeted therapies available and there are many barriers to a pharmacological approach to treatment.

The challenge is clear, how do we identify and develop effective therapies for all CF individuals? In theory, replacing the mutant *CFTR* sequence with the normal sequence could restore CFTR function regardless of mutation. Generally, this might be achieved by one of three approaches: (1) *in vivo* delivery of normal *CFTR* sequence, e.g., via viral vectors, (2) *in vivo* editing of the mutant *CFTR* sequence or, (3) delivery of cells carrying the normal *CFTR* sequence to replace cells carrying the mutant sequence. In this review, we focus on the approach of cell-based therapy for CF lung disease. Although there are compelling examples of effective cell-based therapies, such as hematopoietic stem cell transplantation, there are many challenges facing such an approach for lung disease. What cell type is best suited to restore CFTR function to the airways? How might cells be effectively but safely delivered to a CF patient’s lungs? Here we will review the most promising cellular candidates to treat CF, human bronchial epithelial cells (HBECs) and induced pluripotent stem cells (iPSCs). Finally, we will discuss the major hurdles facing the field of CF cell-based therapeutics, including delivery and engraftment of cells into a diseased host.

## Overview of Airway Epithelial Biology in CF

**FIGURE 1:**
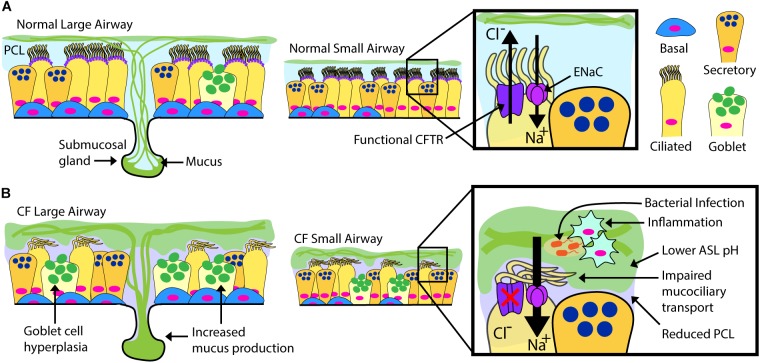
Normal vs. CF Airway. Schematic drawings of normal and CF airways, illustrating selected components of epithelial apical ion transport. **(A)** Secreted mucins are produced by submucosal glands in the large airways and surface goblet cells, forming the overlying, mucin-rich component of the airway surface layer (ASL). This layer is normally transported by effective beating of cilia in the periciliary layer (PCL). ASL hydration is largely controlled by the balance of chloride secretion through CFTR and sodium reabsorption by the epithelial sodium channel (ENaC). **(B)** Mucin overproduction from hypertrophic submucosal glands and hyperplastic surface goblet cells characterize the CF large airways. Non-functional or absent CFTR leads to diminished chloride transport and increased sodium transport through ENaC. Airway dehydration reduces the PCL, impairing mucociliary transport. Accumulation of thick, sticky mucus increases overall ASL thickness and promotes a low pH environment favoring chronic bacterial infection and inflammation. The CF ASL environment is a likely barrier for cell therapy.

Airway epithelial cells, including club, goblet, multi-ciliated, basal and neuroendocrine cells occupy a vital environmental interface. Their normal physiological function is essential to maintain respiratory tract health, and the cells are intimately involved in the pathogenesis of multiple respiratory tract maladies, including the common diseases asthma and chronic obstructive pulmonary disease (Randell et al., 2009). In CF, a dehydrated and acidic ASL impairs critical innate defense mechanisms including mucociliary clearance (Randell et al., 2006; Button et al., 2012; Stoltz et al., 2015). There is current debate on the airway cell types that express *CFTR*, but it is detected in the submucosal glands (Jiang and Engelhardt, 1998) of the airway and in certain surface epithelial cells including multi-ciliated cells (low expression) and in the recently described pulmonary ionocyte (high expression) (Jiang and Engelhardt, 1998; Montoro et al., 2018; Plasschaert et al., 2018). There is debate whether cell autonomous loss of CFTR is hyper-inflammatory *per se*, but the combination of mucus stasis and chronic infection clearly results in highly inflamed CF airways (Roesch et al., 2018). Complex interactions during infection stemming from the epithelial defect ultimately cascade into pathologic changes including extensive mucus obstruction, ectasis of bronchi and bronchioles, and consequent loss of pulmonary function (Figure 1). It is likely that significant structural damage to the airways and surrounding parenchyma becomes irreversible, and a goal for airway epithelial cell therapy is to intervene early, likely in childhood.

## Human Bronchial and Bronchiolar Epithelial Cells (HBECs)

Epithelial cell proliferation in the lung is relatively quiescent at steady state, but this organ retains the ability to regenerate rapidly after injury (Hegab et al., 2012). Much has been learned in recent years about lung stem cells and their roles in repair and regeneration, with particular emphasis on the alveolar parenchyma (Hogan et al., 2014; Kotton and Morrisey, 2014). Cell-based therapy for CF will logically be focused on the conducting airway epithelium, the primary site for mucus stasis, infection, and inflammation. The airways harbor distinct cell populations along the proximal to distal axis that exhibit self-renewal and differentiation capacity. Here, we propose that HBECs may serve as candidates for autologous cell therapy of CF. We review the stem cell properties of HBECs with a focus on the unique advantages and challenges posed for therapeutic translation.

## Stem Cells in the Conducting Airways

The human conducting airways are composed of a pseudostratified epithelium extending from the trachea to the proximal end of small bronchioles. The predominant evidence is that basal cells are the stem cells in this region (Rock et al., 2009). Basal cells are relatively undifferentiated and express a number of classic markers including *TP63* and *KRT5.* The regenerative capacity of basal cells has been extensively demonstrated through *in vitro* clonal growth assays, tracheal graft regeneration, and *in vivo* lineage tracing studies (Gray et al., 1991; Liu et al., 1994; Hong et al., 2004; Rock et al., 2009). Basal cells have the capacity to differentiate into the major cell types of the conducting airways including club (previously Clara) cells (Schoch et al., 2004; Rock et al., 2009), pulmonary neuroendocrine cells (Montoro et al., 2018), the recently described pulmonary ionocyte (Montoro et al., 2018; Plasschaert et al., 2018), and serous and mucus cells in the glands (Hegab et al., 2012, 2014). It is thought that ciliated and goblet cells are generated through a club cell intermediate (Tata et al., 2013; Widdicombe and Wine, 2015), but recent single cell mRNA sequencing studies also suggests direct differentiation of basal cells into ciliated cells (Plasschaert et al., 2018). Basal cell differentiation into pulmonary ionocytes and ciliated cells (either directly or indirectly) is of particular interest for CF cell therapy as these cell types express CFTR and are primarily responsible for epithelial ion transport (Rock et al., 2009; Montoro et al., 2018). The ability of lung basal cells to self-renew and replace luminal CFTR-expressing cell types is vital for cell therapy to be long-lasting.

The basal cell population is likely heterogeneous, containing subpopulations with different proliferation and differentiation capacities. At steady state, basal cell subsets express early differentiation markers for club or ciliated cells including the intracellular domain of Notch2 (N2ICD) or the proto-oncogene transcription factor MYB (C-Myb), respectively, suggesting subpopulations already primed for differentiation (Pardo-Saganta et al., 2015; Watson et al., 2015). Mechanisms regulating lung cell development, homeostatic maintenance, and repair have been recently reviewed (Lee and Rawlins, 2018). However, there is still debate about which cell lineage model is most applicable to the human pseudostratified airway epithelium. Are there rare populations of stem cells in specific niches that undergo asymmetric divisions, generating committed downstream progenitors, or is the stem cell compartment more widely distributed and stochastically regulated? While cell harvesting, gene correction, expansion, and delivery of intermediate progenitors may be efficacious, it is logical that starting with cells exhibiting extensive growth potential and differentiation capacity would be advantageous. Thus, identifying basal cell subsets and gene signatures corresponding to high clonal growth capacity remains an important goal that will theoretically serve as the basis for efficacious CF cell therapy.

Additional cell types within the airways should be considered as alternative candidates to restore CFTR function to the epithelium. Columnar epithelial cells from the pseudostratified regions demonstrate extensive plasticity in which they can dedifferentiate into basal cells (Tata and Rajagopal, 2017). Studies in the porcine model of CF suggest the importance of submucosal gland dysfunction in CF pathogenesis (Hoegger et al., 2014) and lineage tracing in mice illustrates a key role for gland myoepithelial cells in repair (Lynch et al., 2018; Tata et al., 2018). The ability to access, propagate and correct human gland myoepithelial cells, and their ultimate return to gland tubular and acinar niches will require more study.

## Obtaining, Expanding, and Gene Correcting HBECs

Conceptually, HBECs can be obtained from the lungs of CF individuals using one of three minimally invasive techniques, induced sputum sample collection, bronchiolar lavage, and endobronchial brushing or biopsy. These methods yield approximately 2 × 10^3^, 5.5 × 10^3^, and 2 × 10^6^ cells, respectively (Mou et al., 2012; Pollock et al., 2013; Butler et al., 2016). Considering how few cells effectively engraft by current approaches, the paucity of HBEC starting materials poses a considerable challenge to their use in cell therapy. As such, autologous cell-based therapy for CF with somatic epithelial cells will likely require *in vitro* culture for both cell expansion and gene correction. Fortunately, there is a long history of cell culture techniques to expand and subsequently differentiate a predominantly basal cell population from mouse and human large airways (see Table 1 and Bove and Randell, 2015). Conventional expansion methods can be augmented to increase population doublings using dual SMAD inhibition or Rho kinase inhibition along with irradiated mouse 3T3-J2 fibroblasts co-culture (Suprynowicz et al., 2012; Mou et al., 2016). Key reagents such as irradiated 3T3-J2 feeder cells and irradiated serum developed for keratinocytes have been approved for clinical applications by some regulatory authorities and can be, similarly, employed in modern protocols to expand primary HBECs (Suprynowicz et al., 2012; Gentzsch et al., 2017).

**Table 1:** Key studies in the development of culture methods for HBECs and directed differentiation protocols to derive airway epithelial cells from iPSCs.

Human bronchial epithelial cell culture methods	Reference
Clonal growth of human bronchial epithelial cells	Lechner et al., 1981
Detailed description of a media for proliferation of human bronchial epithelial cells on plastic	Lechner and LaVeck, 1985
Differentiation of serially passaged cells at an air-liquid interface (ALI)	Gray et al., 1996
Current detailed methods for ALI cultures using proprietary reagents	Neuberger et al., 2011
Current detailed methods for ALI cultures using non-proprietary reagents	Fulcher and Randell, 2013
Detailed methods for Conditionally Reprogrammed Cells (CRC, with feeder cells)	Gentzsch et al., 2017; Liu et al., 2017
Dual SMAD inhibition method for cell expansion	Mou et al., 2016
Method allowing clonal expansion of CRC primary HBECs	Peters-Hall et al., 2018
CRC method with proprietary media and no feeder cells	Zhang et al., 2018
**Derivation of human airway epithelial cells from iPSCs**	
TGFβ/BMP inhibition required to generate anterior foregut endoderm from iPSC	Green et al., 2011
Differentiation of human CF iPSCs into airway epithelial cells	Mou et al., 2012
Efficient differentiation of human iPSCs into lung epithelial cells comprised of both proximal and distal epithelial cells	Huang et al., 2013
Generation of multiciliated cells from human iPSCs	Firth et al., 2014
Human iPSC-derived lung organoids composed of epithelium and mesoderm	Dye et al., 2015
Surface marker sort method (Carboxypeptidase M) and culture conditions to generate neuroendocrine and functional multiciliated cells	Konishi et al., 2016
Fluorescent lineage reporters to purify early lung progenitors and generate epithelial-only organoid	Hawkins et al., 2017
Wnt withdrawal after early lung specification required for airway patterning	McCauley et al., 2017
Engraftment of iPSC-derived similar to embryonic lung-tips into immunocompromised mouse lungs	Miller et al., 2018


While the larger airways are lined by a tall pseudostratified epithelium the more distal bronchi and bronchioles are lined by a progressively shorter epithelium, in which basal cells eventually extinguish. In the last generations of the small airways, the epithelium becomes simple columnar to cuboidal where stem cells are thought to be contained in the club cell compartment, with an independent lineage existing among pulmonary neuroendocrine cells (Hogan et al., 2014), although pulmonary neuroendocrine cell generation of club and ciliated cells has been observed (Song et al., 2012). This distal region is an important target since small airway obstruction and air trapping is thought to be a defining feature of early CF (Rosenow et al., 2017). Culture methods for human small airway epithelial cells are less well developed than for the large airway, and cells of the smallest bronchioles appear to be more dependent on mesenchymal signals (Lee et al., 2017; Zepp et al., 2017). Specific efforts to better understand human small airway stem cells, their propagation, and eventual engraftment are required.

There have been tremendous advances in genome editing techniques in the last decade, including zinc-finger nucleases, transcription activator-like effector nucleases (TALENs), and the clustered regularly interspaced short palindromic repeats (CRISPR)/Cas9 technique (Zhang et al., 2014). While of utmost importance for correcting a monogenic but complex lung disease such as CF, optimal methods for *CFTR* gene correction of HBECs with minimal off target, potentially carcinogenic risks, followed by cell expansion that maintains long-lived basal stem cells remain to be determined.

## Induced Pluripotent Stem Cells (iPSCs)

iPSCs have several features relevant to their suitability as a source for cell-based therapies. Pluripotency refers to the capacity to differentiate into all cell types in the body. iPSCs can now be routinely generated from any human while retaining an individual’s unique genetic information, without the need for an invasive procedure. The capacity of these cells to proliferate while retaining pluripotency means sufficient numbers for cell-based therapy might someday be feasible. In the following section we review the essential studies that made the discovery of iPSCs possible, summarize the recent progress in deriving lung epithelial cells from iPSCs (Table 1), particularly cell types relevant to CF, and finally highlight the many challenges to be addressed before these cells can be transplanted into CF recipients.

## History of iPSCs

In 2006, Takahashi and Yamanaka made the remarkable discovery that the overexpression of four key factors in somatic cells could “re-program” cells into iPSCs (Takahashi and Yamanaka, 2006; Takahashi et al., 2007). This breakthrough was made possible by decades of prior work in the field of embryonic stem cells (ESCs) that identified: (1) the transient presence of a population of pluripotent stem cells in the developing embryo, (2) the culture conditions sufficient to capture, *in vitro* expand, and maintain the pluripotency of cells isolated from early embryos, and (3) the gene-regulatory networks that control the ground-state of pluripotency (Solter et al., 1970; Stevens, 1970; Evans and Kaufman, 1981; Smith, 2001). Given the regenerative potential of ESCs there was much initial enthusiasm for their therapeutic promise but also controversy over the ethical and moral considerations regarding the use of fetal tissue. From a scientific perspective the main drawback for using ESCs as a cell-based therapy was that even if the cell type of interest could be generated efficiently and safely, the cells would be allogeneic to the recipient and the issues of rejection and immunosuppression that limit the effectiveness of solid organ transplants would persist. Dr. Jim Gurdon’s pioneering nuclear transfer experiments in the 1960s first suggested the feasibility of reprogramming a somatic cell into an autologous pluripotent stem cell (Gurdon, 1962). When a frog egg nucleus was replaced with an intestinal cell nucleus a viable tadpole was produced confirming that the cellular identity could be “reset” to a pluripotent state. Takahashi and Yamanaka set about screening candidate factors that might have the same effect. They identified four factors, *Oct4*, *Sox2*, *Klf4*, and *c-Myc* that were sufficient to reprogram mouse somatic cells (initially skin fibroblasts) into pluripotent stem cells. These “induced” pluripotent stem cells were virtually indistinguishable from ESCs based on their similar transcriptional profiles and capacity to differentiate into all three germ layers when injected subcutaneously in a teratoma assay. Remarkably, iPSCs also contributed to embryogenesis when injected into a blastocyst. One year later, the same four factors were confirmed to reprogram human fibroblasts into iPSCs. Crucial to the emerging field of regenerative medicine, iPSCs have four key characteristics: (1) they are generated from the potential recipient, i.e., autologous, (2) they provide an unlimited supply of pluripotent stem cells without the need for embryonic tissues, (3) they are amenable to gene correction approaches, and (4) they are now routinely derived.

## Directed Differentiation of iPSCs Into Lung Epithelium

Over the past decade, many groups contributed to major progress toward generating lung epithelial cells from iPSCs (Table 1). Directed differentiation refers to the approach of recapitulating, *in vitro*, the key embryonic developmental milestones that lead to the specification of the cell(s) of interest. This is typically achieved through the stepwise addition of signaling factors identified in mouse studies of organogenesis. The field of lung directed differentiation has evolved from generating relatively immature lung epithelial cells to recent reports of increasingly sophisticated protocols that produce relatively mature and functional lung epithelial cells that are currently being applied to lung disease modeling, drug screening and the first forays into *in vivo* transplantation.

In order to understand the directed differentiation process, a brief overview of lung development is first required. The lung develops from the foregut endoderm and is first identified by the expression of *Nkx2-1*, a key transcription factor. *Nkx2-1*is first detected at E9.0 in mice and day 28 in human embryos (Lazzaro et al., 1991; Morrisey and Hogan, 2010). During the first stages of lung organogenesis *Nkx2-1^+^* cells form two primary lung buds and the adjacent foregut separates into two tubes, a ventral trachea and dorsal esophagus. During the pseudoglandular stage, the lung buds undergo branching morphogenesis and proximal-distal patterning. This process, controlled by reciprocal signaling between the lung epithelium and surrounding mesenchyme, gives rise to a highly branched airway tree composed of basal, secretory, multi-ciliated and neuroendocrine cells and an alveolar compartment composed of types 1 and 2 pneumocytes. A number of signaling pathways are involved in the epithelial-mesenchymal interactions that control these stages of lung development including SHH, WNTs, FGFs, retinoic acid and BMPs (Hawkins et al., 2016).

To differentiate iPSCs into lung epithelial cells researchers seek to reproduce these key developmental events *in vitro*. The first major developmental stage in the road map of lung development is the formation of definitive endoderm. Mimicking the *in vivo* patterning of the primitive streak, the addition of the Nodal agonist Activin-A to iPSCs or ESCs produces a highly enriched population of endodermal cells that express classic endoderm markers (including *FOXA2* and *SOX17*) (Kubo et al., 2004; D’Amour et al., 2005; Christodoulou et al., 2011). The protocols to generate definitive endoderm are now well established and form the basis of all endodermal differentiation protocols including lung, liver, pancreas and intestine. The next important developmental step is directing definitive endodermal cells (*FOXA2^+^*) toward an anterior-foregut fate (*FOXA2^+^/SOX2^+^*), analogous to the anterior-posterior axis patterning in the primitive gut tube. The field of lung directed differentiation is relatively new and not surprisingly the published protocols from different groups vary significantly in terms of the timing and combination of growth factors and inhibitors applied at different stages. For example, differing approaches have achieved *in vitro* foregut patterning. In one method antagonism of BMP and TGFβ pathways after endoderm induction was identified as the most potent condition to maintain *FOXA2* expression, and to induce the anterior endoderm marker *SOX2* while suppressing a hindgut fate that expresses *CDX2* (Green et al., 2011). The inclusion of BMP and/or TGFβ inhibition has been adopted by others (Longmire et al., 2012; Mou et al., 2012; Firth et al., 2014; Dye et al., 2015; Hawkins et al., 2017). Alternative approaches include the combination of FGF2 and SHH signaling (Wong et al., 2012). Lung specification is the next major developmental milestone and is indicated by the expression of *NKX2-1* in foregut endoderm cells. Several groups have concluded that WNT, through recombinant WNT3a or glycogen synthase kinase 3 inhibitors, BMP4, and retinoic acid pathway activation are required for lung specification (Huang et al., 2013; Gotoh et al., 2014; Hawkins et al., 2017). Others have developed quite different approaches that led to specification of a lung program, for example using a combination of BMP and TGFβ inhibition, plus SHH and WNT activation (Dye et al., 2015). Early reports demonstrated lung competence of these cells via upregulation of differentiation markers over time. However, both proximal and distal lung markers were frequently present suggesting that the precise signals patterning lung progenitors toward proximal vs. distal fates and to specific differentiated cell types are incompletely known. Competence of these cells to differentiate into functional airway lineages was demonstrated by transitioning to air-liquid interface conditions used to differentiate primary HBECs, or by injecting the cells into the flank or kidney capsule of immune-deficient mice (Wong et al., 2012; Huang et al., 2013; Firth et al., 2014). More recently, there has been progress in generating lung organoids from iPSC-derived lung epithelium, reminiscent of the 3-D organization of lung tissue (Gotoh et al., 2014; Dye et al., 2015; Chen et al., 2017; Hawkins et al., 2017; Miller et al., 2018). In some cases, these protocols involve the co-development of mesodermal and endodermal compartments. To overcome the heterogeneity of cell types produced in these protocols, fluorescent lineage reporters or surface markers have been employed to prospectively isolate the lung epithelial cells of interest. Surface markers including Carboxypeptidase M or the combination of CD47^hi^/CD26^lo^ were identified to isolate immature *NKX2-1^+^* lung epithelial cells and to generate lung epithelial organoids depleted of other endodermal and mesodermal cell types (Gotoh et al., 2014; Hawkins et al., 2017). Although less relevant as a cell-based therapy for CF, it is worth highlighting some of the most compelling progress toward generating relatively mature, functional type 2 pneumocytes from iPSCs. Two groups, adopting the approach of targeting fluorescent reporters to the endogenous *SFTPC* locus, developed similar protocols to isolate and propagate long term a population of *SFTPC^+^* cells highly reminiscent of alveolar type 2 cells (Jacob et al., 2017; Yamamoto et al., 2017).

## Directed Differentiation of iPSCs Into Airway Epithelium

To cure CF airway disease, a cell-based therapy would ideally replace the CFTR-expressing columnar luminal epithelial cells, including terminally differentiated multi-ciliated cells and perhaps ionocytes, but also the stem/progenitor cells that maintain the luminal cells. A number of groups have demonstrated the feasibility of generating CFTR expressing airway epithelium composed of multi-ciliated cells and cells reminiscent of basal and secretory cells (Wong et al., 2012; Firth et al., 2014; Konishi et al., 2016; McCauley et al., 2017). These airway epithelial cells broadly recapitulate the CFTR dysfunction seen in the primary cells of CF patients when interrogated using established CFTR functional assays including ion transport in Ussing chambers and forskolin-induced spheroid swelling. In an important proof-of-concept study, Crane et al., demonstrated the correction of the F508del mutation in iPSCs using zinc-finger nucleases and restoration of CFTR function in those cells after lung differentiation (Crane et al., 2015). Based on our current knowledge, an ideal iPSC-derived candidate cell for engraftment into CF airways is the airway basal cell. A number of groups have described the presence of a subset of basal-like cells after directed lung differentiation as evidenced by the expression of key basal cell markers (e.g., *TP63*, *KRT5*) (Dye et al., 2015; Konishi et al., 2016; Chen et al., 2017; Hawkins et al., 2017; McCauley et al., 2017). However, the efficient derivation of bonafide basal cells, as evidenced by transcriptional profiling and functional demonstration of differentiation into multi-ciliated and secretory cells comparable to those generated by primary basal cell controls, has not yet been convincingly achieved.

## *In Vivo* Engraftment of iPSC-Derived Lung Epithelial Cells

Provided that it is possible to derive a candidate cell type *in vitro*, would that cell engraft and function once transplanted? This is a major question now facing this field and very little is known about the *in vivo* potential of iPSC-derived cells in the lung. Recently, the first report of human iPSC-derived lung epithelial cells engrafting in a murine lung injury model was described (Miller et al., 2018). Miller et al., identified conditions to capture and expand cells with a transcriptional profile similar to the multipotent cells of the developing lung bud tip. The iPSC-derived lung tip cells were delivered intra-tracheally into immunocompromised mice following airway injury with naphthalene. Engrafted cells persisted for up to 6 weeks after transplantation and these cells had adopted a predominantly airway and mostly secretory cell fate with rare multi-ciliated cells. This study is an important first step establishing the feasibility of using animal models to develop engraftment strategies, and suggests that the micro-environment or niche may play an important role in the cell fate decisions of transplanted cells.

## Challenges Facing iPSCs as a Cell-Based Therapy

Despite the progress made in the field of iPSC directed differentiation into lung cells, there are major challenges facing the application of cell-based therapy for the treatment of CF. To date, the derivation of a pure population of airway epithelial cells from iPSCs in sufficient numbers to engraft in human airways has not yet been achieved. How will adequate numbers of differentiated cells be generated for patients? How safe are iPSC-derived lung epithelial cells? The same factors and culture conditions that allow for the unimpeded self-renewal and pluripotency also predispose to tumorigenicity; in fact, a survey of the exomes of 117 human iPSC lines identified nine with mutations in the tumor suppressor gene, *TP53*, with evidence that iPSCs with the mutation had a survival advantage (Ben-David and Benvenisty, 2011; Merkle et al., 2017). Other groups have confirmed that gene editing with CRISPR/Cas9 selects against cells with a normal p53 pathway (Haapaniemi et al., 2018; Ihry et al., 2018). Initially iPSCs were generated using viral vectors that randomly integrated into the genome (Takahashi and Yamanaka, 2006). This approach raised obvious concerns about the malignant potential of iPSCs if an important tumor suppressor locus was disrupted. A non-integrating episomal-based method, such as Sendai virus, is a more logical approach to derive clinical-grade iPSCs (Fusaki et al., 2009). The delivery of safe cells will require a robust and reproducible process to generate iPSCs and differentiated cells, compliant with relevant current good manufacturing practice (cGMP) and regulatory agency approval. Confirmation that the resulting cells are free of integrations and somatic mutations will be needed (Baghbaderani et al., 2015). Transplanted cells will require *in vitro* gene-correction to express functional CFTR. With reprogramming and passaging, and additional time needed to gene-edit and expand cells, the probability of somatic mutations will increase. Furthermore, the process of gene-editing brings additional risk of off-target double-stranded breaks with the potential of disrupting other genetic loci (Zhang et al., 2015). As the goal of cell-based therapy is to replace diseased endogenous lung epithelium with engineered cells, it will be essential to generate pure populations of lung epithelial cells that are highly similar to their *in vivo* counterparts. The field is relatively new and most reports have not focused on the non-lung cells that might also be produced during the differentiation process. For example, an iPSC line expressing GFP from the NKX2-1 locus (*NKX2-1*^GFP^) identified significant variability in the percentage of GFP*^+^* cells, and analysis of the GFP*^-^* population confirmed expression of liver and intestine markers (Hawkins et al., 2017; Serra et al., 2017). Even after a purification step to sort *NKX2-1^GFP+^* cells, McCauley et al. applied single-cell RNA sequencing to airway and alveolar iPSC-derived organoids and identified distinct populations of non-lung endoderm including hepatic-like and gastric-like cells(McCauley et al., 2018). The quest to generate pure populations of well-defined lung epithelial cells will continue and be aided by powerful new techniques such as single-cell RNA sequencing and expanding knowledge of the transcriptional profile and signaling pathways involved in human lung development (Treutlein et al., 2014; Nikolić et al., 2017; Miller et al., 2018). The appropriate number of donor cells required to repopulate a sufficient portion of the diseased airway epithelium is unknown. CFTR levels 15–20% of normal airway expression may be enough to effectively treat CF airway disease (McKone et al., 2003). Building on these aforementioned advances, the ultimate final hurdle to a cell-based therapeutic approach for CF is engraftment.

## Engraftment

**FIGURE 2:**
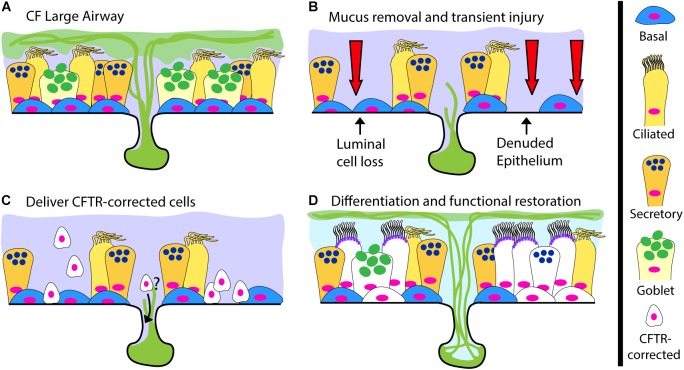
A Cell-Based Therapy Model for CF. **(A)** The pre-treatment CF large airway. **(B–D)** A depiction of cell-based therapy in three steps: **(B)** Mucus removal and transient preconditioning of the airway epithelium, potentially requiring luminal epithelial surface cell loss and partial epithelial denudation; **(C)** Delivery of CFTR-corrected stem and progenitor cells to the surface epithelium and possibly the submucosal glands; **(D)** Differentiation of delivered CFTR-corrected cells into all relevant cell types, restoration of functional CFTR ion-transport and a return to normal airway physiology.

The gold standard for cell-based therapy is hematopoietic cell transplantation. This now commonplace and life-saving procedure relies on delivering hematopoietic progenitor cells after inducing tissue receptivity through marrow-ablative doses of cytotoxic agents and radiation. The term engraftment is used to describe the reconstitution of host bone marrow by donor cells. For CF, successful airway engraftment is thought of in similar terms, recipient airway priming, donor cell survival and regeneration of the airway epithelium (Figure 2). The ideal strategy to deliver a cell-based therapy for CF, or any lung disease, has yet to be developed and the prospect of deliberately injuring the lungs of CF recipients to ablate endogenous cells is daunting. An ideal engraftment strategy would involve the minimal airway injury necessary, in particular to the small airways, to promote a microenvironment conducive to long-lived engraftment of cells in numbers sufficient to restore CFTR function. There are many hurdles the scientific community must overcome to achieve human airway engraftment such as determining: (1) the most suitable cell type(s), (2) the methods to safely scale-up cell production, (3) the optimal injury, (4) supportive measures for oxygenation/ventilation and (5) methods to control mucus and infection to allow engraftment of cells. Despite these challenges, there are promising reports of effective cell therapies for other organ systems that support the overall feasibility of this approach. Based on pioneering work from the laboratory of Howard Green, autologous epidermal keratinocytes have been extensively evaluated as treatment for burns (Green et al., 1979; Ter Horst et al., 2018). Recently, transgenic autologous keratinocytes with a correct copy of a gene mutated in the blistering skin disease junctional epidermolysis bullosa were used to replace nearly the entire epidermis of a critically ill child with 60% skin loss, miraculously allowing hospital discharge and return to school (Hirsch et al., 2017). This remarkable achievement not only alleviated patient suffering, but also provided vital insights about epidermal keratinocyte stem cell dynamics, indicating the importance of long-lived stem cells for epidermal restoration. Additional experience comes from corneal limbal epithelial stem cell transplantation in which damage to one eye can be treated with autologous stem cells from the contralateral eye (Rama et al., 2017; Sasamoto et al., 2018). In the lung field, a number of groups have recently reported evidence of lung engraftment in animal models (Rosen et al., 2015; Vaughan et al., 2015;Ghosh et al., 2017; Nichane et al., 2017; Ma et al., 2018; Miller et al., 2018). Here we will briefly review this progress and highlight important next steps.

A number of injury models, including naphthalene, sulfur dioxide, radiation and detergents, have been developed to study the biology of the airway epithelium (Lamb and Reid, 1968; Plopper et al., 1992; Borthwick et al., 2001; Leblond et al., 2009; Rosen et al., 2015). It is unclear to what extent, if any, these models might be used in humans but they have provided a platform to study and develop engraftment strategies. Embryonic lung, both mouse and human, contain stem/progenitor populations with engraftment capacity (Rosen et al., 2015; Nichane et al., 2017). In one approach, intravenous administration of a single cell suspension of whole canalicular stage lung (mouse E15-16 or human week 20–22) into mice pre-conditioned with both naphthalene and total body irradiation, resulted in epithelial, mesenchymal, and endothelial patches of engrafted cells (Rosen et al., 2015). Higher rates of chimerism were detected in mouse-into-mouse compared to human-into-mouse experiments. It is surprising that intravenous delivery of a mixture of embryonic lung cell types resulted in homing of cells to their unique anatomic compartments. Further work is required to understand the safety of this approach including the consequence of cells being delivered to the wrong compartment within the lung or even to other organs. In terms of an approach for CF, the majority of epithelial engrafted cells had an alveolar phenotype although some airway-like cells were detected and some expressed CFTR. Interestingly, the co-administration of mesenchymal and endothelial cells was associated with improved epithelial cell engraftment. The multitude of practical, ethical and political issues surrounding the use of fetal tissue make this an unlikely future option for cell-based therapy. However, iPSCs may provide access to autologous cells at embryonic time points with capacity to engraft (Miller et al., 2018). Adult airway epithelial cells can also engraft in the lung (Vaughan et al., 2015; Ghosh et al., 2017; Nichane et al., 2017). The environment induced by H1N1 influenza infection has garnered much interest for its engraftment potential (Kumar et al., 2011; Vaughan et al., 2015). The viral infection causes widespread damage to the airway and alveolar epithelium and activates rare immature (*TP63^+^/KRT5^-^*) basal cells in the small airways (Yang et al., 2018). Orthotopic transplantation of a variety of airway epithelial cells into influenza-injured mice led to varying levels of engraftment (Vaughan et al., 2015). Perhaps most relevant to the CF field is a recent report of successful engraftment of primary airway basal cells into naphthalene injured mice (Ghosh et al., 2017). Transplanted mouse or human basal cells reconstituted patches of epithelium in the trachea and small airways with evidence of multi-lineage differentiation into secretory and multi-ciliated cells. Themes common to each of the above experiments include: (1) lung injury is required for engraftment of donor cells, (2) different injury models likely result in unique regenerative niches and (3) successful strategies to date suggest multipotent input donor cells are the most promising candidates for engraftment. Looking forward, a number of approaches might accelerate the field of cell-based therapy for lung disease. Engraftment after hematopoietic cell transplantation is monitored by sampling the peripheral blood. Assessing lung engraftment is far more challenging. Methods to interrogate whether donor cells are functionally integrated into the airway epithelium will be essential. Animal studies to optimize injury models and the efficiency, function and safety of engrafted cells are necessary, but are rate-limiting due to low throughput, particularly in larger animals. Developing non-lethal experimental methods, including targeted airway injury and bioluminescent reporter cell lines to track engrafted cells in real-time without the need for necropsy would increase the efficiency of animal engraftment studies.

Methods to incite a targeted, clinically tolerable airway epithelial injury have been tested in humans albeit not in the context of cell-based therapy. Bronchial thermoplasty, an FDA-approved treatment for refractory severe persistent asthma, delivers brief thermal energy to the airways via flexible bronchoscopy and leads to initial epithelial sloughing and a prolonged decrease in smooth muscle with improvement in asthma severity (Miller et al., 2005). In a small pilot study, airway delivery of a metered liquid nitrogen cryospray via bronchoscopy resulted in airway epithelial damage followed by regeneration (Slebos et al., 2017). An alternative approach, with potential for clinical translation, is targeted airway de-epithelialization achieved by instilling mild detergents intra-tracheally (Dorrello et al., 2017). Despite the many limitations of these approaches in the context of CF, including the small size and large number of airways needed to treat CF, they provide preliminary support for the feasibility of a human injury platform. The situation is further complicated for targeting CF in more advanced disease where successful cell-based therapy must also overcome a highly inflamed, architecturally distorted organ characterized by thick mucus and superinfection. Similar to the blood product and growth factor support necessary after hematopoietic cell transplant, lung engraftment strategies will likely require lung supportive measures. The advances and more widespread use of extra-corporeal membrane oxygenation (ECMO) provides a means of supporting oxygenation during respiratory failure (Squiers et al., 2016). Advances in the technology to maintain human lungs using *ex vivo* lung perfusion (EVLP) may provide insights into supporting the injured and regenerating lung (Makdisi et al., 2017). Future approaches for cell-based therapy incorporating these evolving technologies may be necessary. Alternatively, devising injury-independent strategies to promote lung engraftment of therapeutic cells may be an avenue for future research and development.

## Conclusion

To date, CF remains the most common life-limiting hereditary lung disease. Decades of work resulted in important strides to understand molecular mechanisms in CF and treatment options for many patients. Despite this progress, a subset of patients lack effective treatment options and cell-based therapies could theoretically cure CF lung disease. The ideal cell type for this purpose would repopulate the airway epithelium with autologous, functional, and CFTR-expressing cells. Progress in our understanding of airway epithelial biology and endogenous stem/progenitor populations has revealed a number of candidate cell types for airway regeneration, in particular basal cells. The recent revolution in gene-editing techniques means genetic correction of the *CFTR* mutation in basal cells will likely be achieved in the near future and would provide a source of autologous airway stem cells with the normal *CFTR* sequence. In the iPSC field, directed differentiation protocols have evolved from initial studies demonstrating the presence of immature lung-like cells to more recent reports of increasingly efficient protocols to derive functional alveolar and airway epithelial cells. We have detailed some of the major challenges that must be overcome both in terms of generating the cells of interest and engrafting those cells in a safe and effective manner. While there is work yet to be done, there is cautious optimism that a curative cell-based therapy for CF will be achieved in the future.

## Author Contributions

All authors contributed to this manuscript. In collaboration, AB, FH, RL, and SR wrote all sections of this review. RL illustrated the figures. All authors read, edited, and approved of the final version of the manuscript.

## Conflict of Interest Statement

The authors declare that the research was conducted in the absence of any commercial or financial relationships that could be construed as a potential conflict of interest.
